# Clinical Reasoning, Pathophysiology, and Artificial Intelligence in Critical Care: A Conceptual Framework for Integrating Decision-Making

**DOI:** 10.3390/jcm15207987

**Published:** 2026-10-15

**Authors:** Álvaro Réa-Neto, Jeane Cristina Fonseca Vieira

**Affiliations:** 1 Department of Internal Medicine, Federal University of Paraná (UFPR), Curitiba 80060-150, Paraná, Brazil; 2 Post-Graduate Program in Internal Medicine and Health Sciences (PPGMICS), Federal University of Paraná (UFPR), Curitiba 80060-150, Paraná, Brazil; 3 CEPETI—Center for Studies and Research in Intensive Care, Curitiba 82530-200, Paraná, Brazil; jeanefvieira@gmail.com

**Keywords:** medical education, clinical decision support, cognitive bias, diagnostic error, Bayesian reasoning

## Abstract

Medical practice is an interpretive, decision-making activity conducted under uncertainty, and care quality depends on clinicians’ ability to organize knowledge into hypotheses, understand mechanisms, estimate probabilities, and revise decisions as the clinical course evolves. In critical care, where physiology is unstable and trajectories are nonlinear, these demands are magnified. We propose a conceptual framework integrating three complementary dimensions: clinical reasoning, which provides the cognitive architecture of decision-making; pathophysiology, which supplies the causal structure to interpret biological phenomena; and artificial intelligence, which, when well designed and critically used, can organize data, generate hypotheses, and reduce cognitive load. Tracing the evolution of clinical reasoning from the anatomoclinical model to hypothetico-deductive, probabilistic, cognitive, and sociotechnical models, we examine how diagnostic and therapeutic errors arise from interacting cognitive biases, system factors, and case characteristics, and how better problem representation, differential diagnosis, and team culture may mitigate them. We also frame Bayesian reasoning and decision thresholds as the probabilistic grammar of bedside decisions, analyze the risks of artificial intelligence, including automation bias, opacity, and limited metacognition, and propose principles for its safe use. Rather than replacing physicians, artificial intelligence should strengthen clinical reasoning and reinforce calibrated, accountable bedside judgment. These principles are intended as a framework for future research and require empirical validation before clinical translation.

## 1. Introduction

Medicine is a biological science, interpretive practice, and an ethical activity conducted under uncertainty. Physicians rarely make decisions in the presence of complete, stable, and unequivocal information. More often, they decide based on incomplete symptoms, ambiguous signs, imperfect tests, changing probabilities, institutional pressures, patient values, and time constraints. Consequently, the quality of medical practice depends on a clinician’s ability to organize knowledge into plausible hypotheses, recognize patterns, understand mechanisms, estimate probabilities, select proportionate interventions, and revise decisions when the clinical course contradicts the initial interpretation. This set of operations constitutes clinical reasoning: a dynamic, probabilistic, and continuously revisable process of diagnostic and therapeutic decision-making [1]. The importance of this topic has become increasingly evident as diagnostic error is now recognized as a major source of preventable harm. A landmark report by the National Academies of Sciences, Engineering, and Medicine consolidated the concept that diagnosis is a continuous process rather than an isolated event. It depends on the interaction among clinical cognition, care systems, communication, technology, and longitudinal follow-up [2]. Recent estimates suggest that diagnostic errors contribute to a substantial burden of death and disability in the United States [3]. Among hospitalized patients who died or required transfer to the Intensive Care Unit (ICU), these diagnostic errors have also been identified with clinically meaningful frequency, often associated with failures in evaluation, data synthesis, and clinical reassessment [4]. Critical care medicine makes this issue particularly visible. In the ICU, reasoning requires a delicate balance: it should be rapid but not rash, analytical but not paralyzing, grounded in mechanisms while remaining sensitive to probabilities, and evidence-informed but individualized. Critically ill patients are characterized by physiologic instability, multiorgan dysfunction, and nonlinear clinical trajectories where multiple interventions occur simultaneously. In this high-stakes environment, small interpretive failures can alter decisions about mechanical ventilation, vasopressors, antimicrobials, anticoagulation, sedation, fluids, dialysis, or limitation of life-sustaining treatment. In the ICU, reasoning does not end with the diagnosis; it persists through every new arterial blood gas, image, culture, hemodynamic shift, or therapeutic response. Despite the maturity of each of these fields, they have largely developed in isolation. The clinical reasoning literature has focused on the cognitive and educational processes underlying diagnostic and therapeutic decisions. Pathophysiology is usually taught and studied as biomedical science, frequently separate from probabilistic bedside reasoning. Research on artificial intelligence (AI) in critical care has concentrated on prediction, classification, and decision support, with persistent questions about external validity, workflow integration, and clinical impact. What remains largely missing is an operational framework that integrates these dimensions: a probabilistic architecture for updating hypotheses, a pathophysiologic grammar for testing their mechanistic coherence, and a critical, governed use of AI as a cognitive adjunct at the bedside. Existing reviews and models typically address one of these components in depth but seldom their interaction in the time-pressure environment of the ICU. Accordingly, in this article we propose a conceptual framework arguing that contemporary medicine, and critical care in particular, should integrate three complementary dimensions: clinical reasoning, pathophysiology, and AI. Clinical reasoning provides the cognitive architecture of decision-making, while pathophysiology provides the causal structure necessary to interpret complex biological phenomena. When thoughtfully designed and critically applied, AI may expand human capacity by organizing data, generating hypotheses, and reducing cognitive load. However, AI does not eliminate the need for robust clinical reasoning. On the contrary, it increases the need for physicians who can determine whether an algorithmic recommendation is biologically coherent, clinically applicable, ethically acceptable, and appropriate for the actual patient. Because this framework is conceptual, we present it as a testable proposal for research and educational innovation rather than as a validated intervention.

**Search Strategy.** This narrative review was conducted by searching PubMed/MEDLINE from inception through 31 July 2026 and the AI-assisted academic search engine Consensus on 31 July 2026. For PubMed/MEDLINE, the following two search strings were used. The first string addressed clinical reasoning and pathophysiology in critical care: (“clinical reasoning”[MeSH] OR “diagnostic reasoning” OR “hypothetico-deductive reasoning” OR “clinical decision making”[MeSH] OR “Bayesian reasoning” OR “decision theory”) AND (“pathophysiology” OR “disease mechanisms”) AND (“critical care”[MeSH] OR “intensive care” OR “critical illness”) AND (English[lang]). The second string addressed artificial intelligence applied to clinical reasoning in critical care: (“artificial intelligence”[MeSH] OR “machine learning”[MeSH] OR “large language models”) AND (“clinical reasoning” OR “diagnostic error” OR “automation bias”) AND (“critical care”[MeSH] OR “intensive care” OR “critical illness”) AND (English[lang]). No restriction to specific commercial AI models was applied; studies evaluating particular models were captured through the broad search terms, in particular “large language models”. No lower publication date limit was applied, because the review includes a historical section on the evolution of clinical reasoning and, therefore, landmark publications from earlier decades were eligible for screening. The first search string identified 17 records, and the second identified 46 records in PubMed/MEDLINE. A supplementary search using Consensus was also performed to ensure breadth of coverage. Unlike PubMed/MEDLINE, Consensus does not natively support Boolean search strings; accordingly, the search was conducted by entering a natural-language instruction that embedded the same Boolean string used for artificial intelligence in PubMed/MEDLINE, restricted to articles published up to July 2026. This supplementary search identified 251 additional records that were screened by title and abstract independently by both authors, and disagreements were resolved by discussion. These searches were used to map the field and ensure breadth of coverage rather than to provide an exhaustive census of all publications; accordingly, no claim of exhaustive screening of all retrieved records is made. Given the narrative and conceptual nature of this review, and the substantial volume of literature on artificial intelligence in critical care, studies were purposively selected when they directly informed the three dimensions of the framework (clinical reasoning, pathophysiology, and artificial intelligence in critical care). This purposive selection was complemented by snowballing: the reference lists of retrieved articles and of previous reviews were screened to identify seminal theoretical works, landmark publications, textbooks, and clinical guidelines, which were included irrespective of publication date. Table 1 summarizes the principal studies informing this review, with their designs, main outcomes, and limitations.

**Eligibility criteria.** Studies were included if they addressed (a) clinical reasoning or diagnostic reasoning, (b) the role of pathophysiology or biomedical knowledge in clinical decision-making, or (c) artificial intelligence applied to clinical reasoning, diagnostic error, or critical care. We prioritized peer-reviewed original research, seminal theoretical works, systematic reviews, and recent high-impact publications. Inclusion was restricted to articles published in English. For the historical dimension of this review, no date restriction was applied: landmark publications identified through snowballing or expert knowledge were eligible regardless of publication date. Exclusion criteria comprised editorials, conference abstracts, non-peer-reviewed preprints, and studies addressing AI or reasoning outside clinical settings. Two exceptions to the stated restrictions were applied deliberately. First, the foundational reference on clinical reasoning by the corresponding author [1] was originally published in Portuguese and was included (through snowballing and expert knowledge) because it anchors the historical and conceptual framing of this review. Second, a small number of studies addressing medical education, simulation, and human–AI interaction in laboratory or classroom settings (e.g., overreliance on AI dialogue systems, cognitive forcing functions) were included to inform the educational and sociotechnical components of the framework; they are not cited as evidence of clinical effectiveness. Because of the narrative nature of the review, no formal risk-of-bias assessment or quantitative synthesis was performed; instead, the literature was organized thematically around the three dimensions of clinical reasoning, pathophysiology, and AI.

## 2. Historical Evolution of Clinical Reasoning: From the 1940s to the Present

The history of clinical reasoning in the twentieth century reflects a fundamental transition from a practice based predominantly on authority, experience, and taxonomy toward a formal science of decision-making. Until the mid-twentieth century, medical education was strongly centered on the anatomoclinical model, which focused on recognizing syndromes, localizing lesions, and correlating signs and symptoms with anatomical abnormalities. While this paradigm provided a necessary foundation for diagnosis, it proved insufficient for the probabilistic uncertainty and decision-making complexity that characterize modern clinical practice [20].

Beginning in the 1940s and 1950s, three movements challenged it: clinical epidemiology and medical statistics, which addressed uncertainty through sensitivity, specificity, predictive values, and likelihood ratios; decision theory, which formalized weighing probabilities against the benefits, harms, and costs; and the cognitive revolution investigated the mental processes of problem solving [20].

The 1970s consolidated these emerging theories. Elstein, Shulman, and Sprafka proposed the hypothetico-deductive model, in which clinicians generate early hypotheses and then seek data to confirm, reject, or reformulate them [20]. Simultaneously, the work of Tversky and Kahneman revealed that human judgment under uncertainty often relies on heuristics, mental shortcuts such as availability, representativeness, and anchoring [21] and Pauker and Kassirer proposed the threshold model, according to which the utility of testing or treating depends on the pretest probability, test performance, intervention risks, and the consequences of withholding treatment [22]. A few years later, the rise in evidence-based medicine (EBM) codified the integration of high-quality evidence with clinical expertise and patient values, reducing practice variability—while introducing an applicability gap, namely the challenge of translating evidence from selected trial populations to complex individual patients [23]. The 1980s and 1990s shifted clinical reasoning research from prescriptive rule-based models toward descriptive accounts of expertise. Norman and Eva established that expert clinical reasoning does not depend on the linear application of rules, but rather on highly organized knowledge networks in which clinical patterns, mechanisms, contexts, and prior experiences are efficiently integrated [6,7]. The concept of the illness script became central: a mental representation that links predisposing factors, pathophysiologic mechanisms, clinical manifestations, expected findings, and typical evolution, later evaluated as formal strategies for teaching clinical reasoning [5]. Since the 2000s, dual-process theory has emerged as a foundational framework for understanding clinical cognition, distinguishing fast, intuitive processes (Type 1) from slow, analytical deliberation (Type 2). In the ICU, Type 1 reasoning enables the rapid recognition of life-threatening patterns, while Type 2 reasoning provides the necessary analytical oversight to reassess initial hypotheses, integrate discordant data, calculate probabilities, consider alternatives, and mitigate the risk of premature closure [24]. The challenge is not the elimination of intuition, but recognizing when it should be subjected to deliberate analysis, particularly under uncertainty, complexity, or unexpected clinical evolution [25].

In critical care, relying solely on pattern recognition is risky because overlapping mechanisms can be masked by ongoing therapies. As generative AI excels at statistical pattern matching, the unique role of the intensivist shifts toward adaptive expertise—leveraging critical thinking, pathophysiologic reasoning, and clinical judgment to manage complex, atypical scenarios [26].

## 3. How Medical Errors Caused by Flawed Clinical Reasoning Occur and Can Be Prevented

Medical error is a multifaceted phenomenon encompassing technical, organizational, and ethical failures. Within this broad spectrum, errors in clinical reasoning, particularly diagnostic errors and errors in therapeutic decision-making, are prominent. A diagnostic error may be defined as a failure to establish an accurate and timely explanation of the patient’s health problem or to communicate that explanation effectively [2]. Its importance is substantial because the diagnosis guides almost all subsequent decisions. Incorrect diagnosis leads to unnecessary treatments, delays in life-saving therapies, and missed opportunities for reversibility, particularly in the time-sensitive environment of the ICU. In practice, reasoning errors are rarely the result of a single isolated failure. Instead, they emerge from the complex interaction among cognitive factors, system factors, and case characteristics. Cognitive factors include biases, insufficient knowledge, poor problem representation, low tolerance of uncertainty, misinterpretation of tests, and overconfidence. These are often exacerbated by system factors, including excessive workload, frequent interruptions, fatigue, fragmentation, inadequate handoffs, and lack of diagnostic feedback. Finally, the diagnostic challenge is compounded by several case-specific factors: atypical clinical presentations, concurrent comorbidities, impaired patient communication, suboptimal diagnostic accuracy, and accelerated disease evolution [2,3,4]. The most relevant cognitive mechanisms include premature closure, anchoring, confirmation bias, availability bias, and search satisficing. Premature closure develops when physicians accept a diagnosis before sufficient supporting evidence or before relevant alternatives have been considered. Anchoring bias emerges when the initial hypothesis or first information received exerts excessive influence on subsequent interpretations. Confirmation bias leads clinicians to preferentially value data supporting the initial hypothesis while discounting discordant findings. Availability bias makes recently encountered or emotionally salient diagnoses seem more likely than they are. Search satisficing occurs when identifying one abnormality reduces motivation to identify others [21,24,25]. In the ICU, these biases take concrete forms. A patient with sepsis and persistent hypoxemia may be misattributed to “worsening ARDS” when fluid overload, right ventricular dysfunction, pulmonary embolism, or ventilator-associated pneumonia are responsible. A hypotensive patient on norepinephrine may be attributed to vasoplegic shock while occult hypovolemia, tamponade, septic myocardial dysfunction, or dynamic hyperinflation are overlooked. An agitated patient may receive additional sedation for presumed hyperactive delirium when pain, hypercapnia, urinary retention, withdrawal, or ventilator asynchrony are the primary causes. The error lies not in pattern recognition, but in failing to reconsider the diagnosis when clinical evolution contradicts it. Prevention requires a combined approach; awareness of biases alone is insufficient. The strategies outlined below are proposed on the basis of their theoretical plausibility and of supportive, but not yet conclusive, evidence; whether they reduce diagnostic error or improve clinical outcomes requires prospective evaluation. The first strategy involves improving problem representation through selective case summarization that incorporates time course, severity, context, risk factors, pertinent negative findings, and incongruities. “An immunosuppressed older adult with acute dyspnea, low-grade fever, hypoxemia disproportionate to the radiographic findings, and elevated D-dimer after prolonged hospitalization” activates different diagnostic hypotheses than simple pneumonia. This strategy is aligned with the cognitive-science literature on expert reasoning [6,7]; however, its incremental benefit in the ICU has not been quantified.

**Figure 1:**
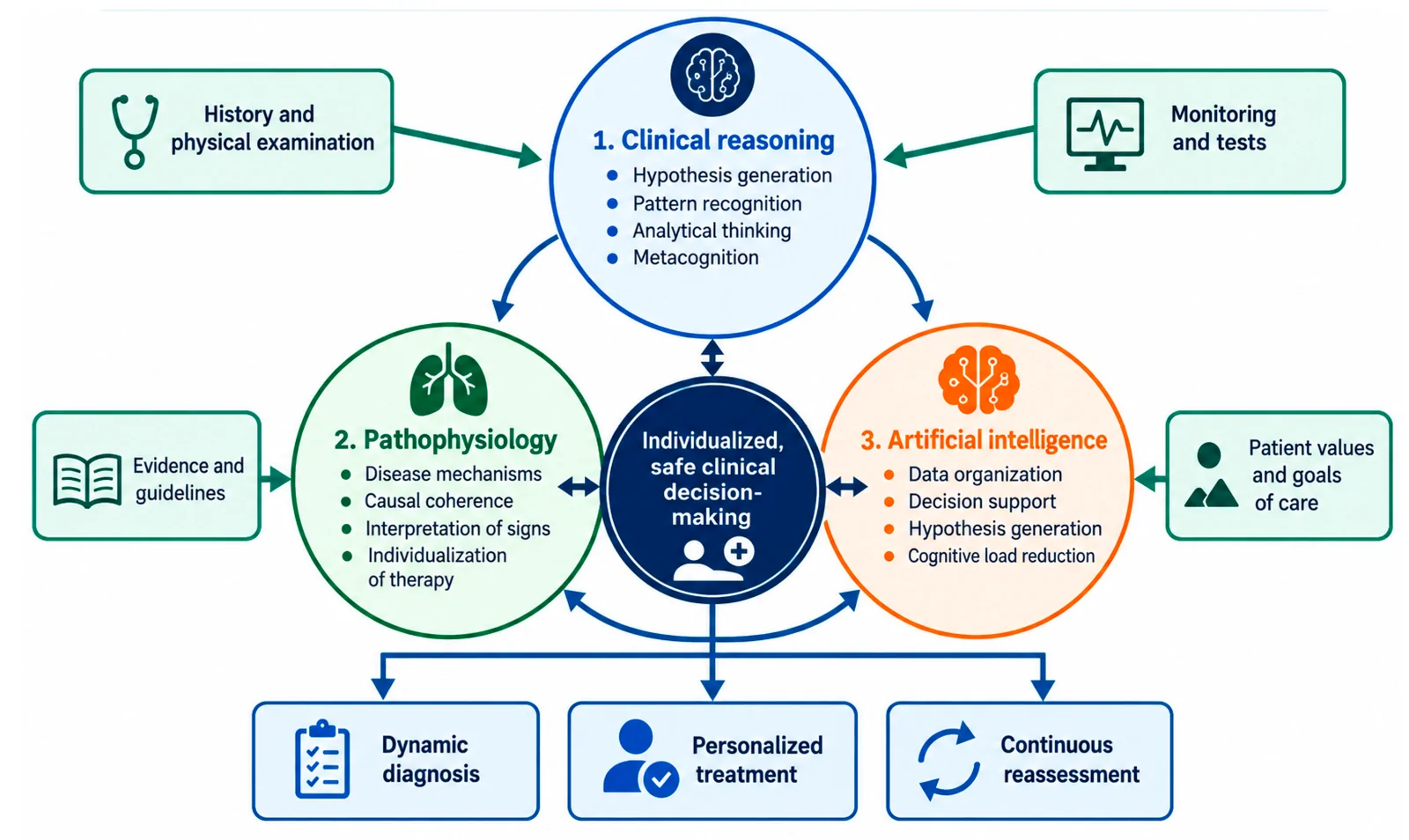
**Integration of clinical reasoning, pathophysiology, and artificial intelligence in intensive care medicine.** This figure illustrates the conceptual integration of three complementary domains that support safe, individualized decision-making in critically ill patients. Clinical reasoning contributes to hypothesis generation, pattern recognition, analytic thinking, and metacognition. Pathophysiology contributes to understanding disease mechanisms, causal coherence, interpretation of clinical signs, and therapeutic individualization. AI contributes to data organization, decision support, hypothesis generation, and reduction in cognitive workload. These three domains interact dynamically and are informed by bedside history and physical examination, monitoring and diagnostic testing, evidence and guidelines, and patient values and goals of care. Their integration supports dynamic diagnosis, personalized treatment, and continuous reassessment. Note: This figure was created with the assistance of ChatGPT GPT-6 (OpenAI), a generative AI tool, based on conceptual prompts designed by the authors.

A second strategy is to maintain an active differential diagnosis. This should not be a ritualistic list, but a hierarchy of hypotheses with probabilities, mechanisms, consequences of missed diagnosis, and tests capable of changing management. In high-risk situations, it is useful to ask: what diagnosis must I not miss? What best explains all findings? Which finding contradicts my leading hypothesis? Which treatment may be masking the presentation? What would change my management in the next hours? This approach is widely taught and conceptually sound, but its isolated contribution to reducing diagnostic error remains to be demonstrated. A third strategy is to incorporate cognitive pauses at critical moments: Admission, unexpected deterioration, therapeutic failure, handoff, invasive decision, or ICU discharge. Three questions may be enough: Does the current diagnosis still explain the clinical course? Is there any new evidence contradicting it? Is there a high-risk alternative cause that must be ruled out now? Cognitive pauses are a promising, hypothesis-generating strategy; no controlled trials have yet demonstrated their effectiveness in improving diagnostic outcomes. A fourth strategy is to value pathophysiology as a coherence test. A hypothesis should explain signs, temporal sequence, and responses to interventions. Pathophysiology serves as a causal framework, distinguishing spurious associations from mechanistically plausible explanations. This is best regarded as a conceptual safeguard: mechanistic plausibility can support a hypothesis, but it does not by itself confirm diagnostic or therapeutic correctness, and its diagnostic value has not been formally evaluated. A fifth strategy is to improve the team’s environment. Many errors are identified by team members who identify incongruities but do not find space to express them; therefore, the question “What are we missing?” should be routine in the ICU. This recommendation is consistent with patient-safety frameworks, although the direct link between team culture and reasoning outcomes remains to be tested (Figure 1).

## 4. Pathophysiology, Disease Mechanisms, and Decision Theory: Impact on the Efficiency of Medical Management

Clinical medicine operates in two complementary languages: diagnostic categories and mechanisms. The first names diseases; the second explains processes such as inflammation, obstruction, vasodilation, hypovolemia, pump failure, shunt, ventilation-perfusion mismatch, mitochondrial dysfunction, and hypercoagulability. Efficient practice depends on both. Diagnoses without mechanisms may lead to generic management; mechanisms without diagnostic categories may lose prognostic, epidemiologic, and therapeutic integration. The literature in medical education has shown that biomedical knowledge is not a dispensable preclinical layer, but an active component of clinical reasoning. Woods argued that it contributes to constructing explanations, transferring learning, and reasoning through difficult cases [27]. Studies of basic and clinical knowledge show that foundational science improves the organization and application of diagnostic knowledge, especially when cases require causal interpretation rather than merely pattern recognition [28]. In typical cases, pathophysiology may remain implicit; in atypical, severe, or contradictory cases, it becomes essential. Pathophysiology improves efficiency by enabling clinicians to select interventions aligned with the predominant mechanism, anticipate adverse effects, recognize paradoxical responses, and individualize treatments. In hypoxemia, identifying whether the main problem is intrapulmonary shunt, hypoventilation, low inspired oxygen fraction, impaired diffusion, or low cardiac output changes the strategy. In shock, recognizing whether vasodilation, hypovolemia, pump failure, or obstruction predominates changes the priority among fluids, vasopressors, inotropes, drainage, thrombolysis, pericardiocentesis, or ventilator adjustment. Respiratory, cardiovascular, renal, neurologic, and metabolic physiology underpin the transformation of data into mechanistic explanations [29,30,31].

In the ICU, mechanistic reasoning is unavoidable. Mechanical ventilation is not merely a treatment for respiratory failure; it alters transpulmonary pressure, venous return, right ventricular afterload, regional distribution of ventilation, and the risk of lung injury. Decisions about PEEP require understanding recruitability, hemodynamics, compliance, and hyperdistention. Decisions about fluid administration require distinguishing fluid responsiveness from net benefit; a patient may respond to fluid and still be harmed by it. The approach to shock illustrates the need to integrate macrohemodynamics, microcirculation, cardiac function, and tissue perfusion [32]. ARDS exemplifies how diagnostic categories must be interpreted mechanistically. The Berlin Definition standardized criteria for timing, imaging, edema origin, and degree of hypoxemia, but it does not replace analysis of recruitability, compliance, dead space, driving pressure, cardiopulmonary interaction, and precipitating etiology [33]. Two patients may meet ARDS criteria and require distinct strategies for ventilation, fluids, prone positioning, sedation, neuromuscular blockade, infectious investigation, or hemodynamic assessment. The same principle applies to sepsis: guidelines provide essential recommendations on early recognition, antimicrobials, source control, resuscitation, and vasopressors. However, safe application requires individualized interpretation of the infectious source, physiologic reserve, and intervention risks [34]. Decision theory complements pathophysiology by emphasizing that clinical decisions are determined not only by mechanisms, but also by probabilities and consequences. Between the testing threshold and the treatment threshold lies a zone in which additional tests are useful. Below the testing threshold, further investigation may cause more harm than benefit; above the treatment threshold, probability and risk justify treatment without waiting for definitive confirmation [22]. This reasoning is routine: starting antibiotics in probable septic shock before a positive culture is available; anticoagulating high-probability pulmonary embolism when delay is dangerous; avoiding computed tomography for clearly benign headache without red flags; or abandoning a hypothesis when multiple findings reduce its plausibility.

Contemporary critical care medicine is moving toward personalization. The same syndromic diagnosis contains distinct subphenotypes. ARDS may result from pneumonia, aspiration, extrapulmonary sepsis, pancreatitis, trauma, or transfusion; it may have greater or lesser recruitability; it may coexist with shock, right ventricular failure, or intra-abdominal hypertension. Sepsis may present hyperinflammatory, immunosuppressed, vasoplegic, hypovolemic, cardiodepressed, or dominated by microcirculatory dysfunction. Recent proposals for personalized medicine based on individualized treatment effects reinforce that the ICU is a privileged environment for quantifying response heterogeneity and adapting interventions to specific physiologic, prognostic, and biological profiles [35]. Multimorbidity, common in older critically ill patients, expands this need because diseases and treatments interact nonlinearly [36].

### Bayesian Reasoning: Continuous Updating of Clinical Probability

**Figure 2:**
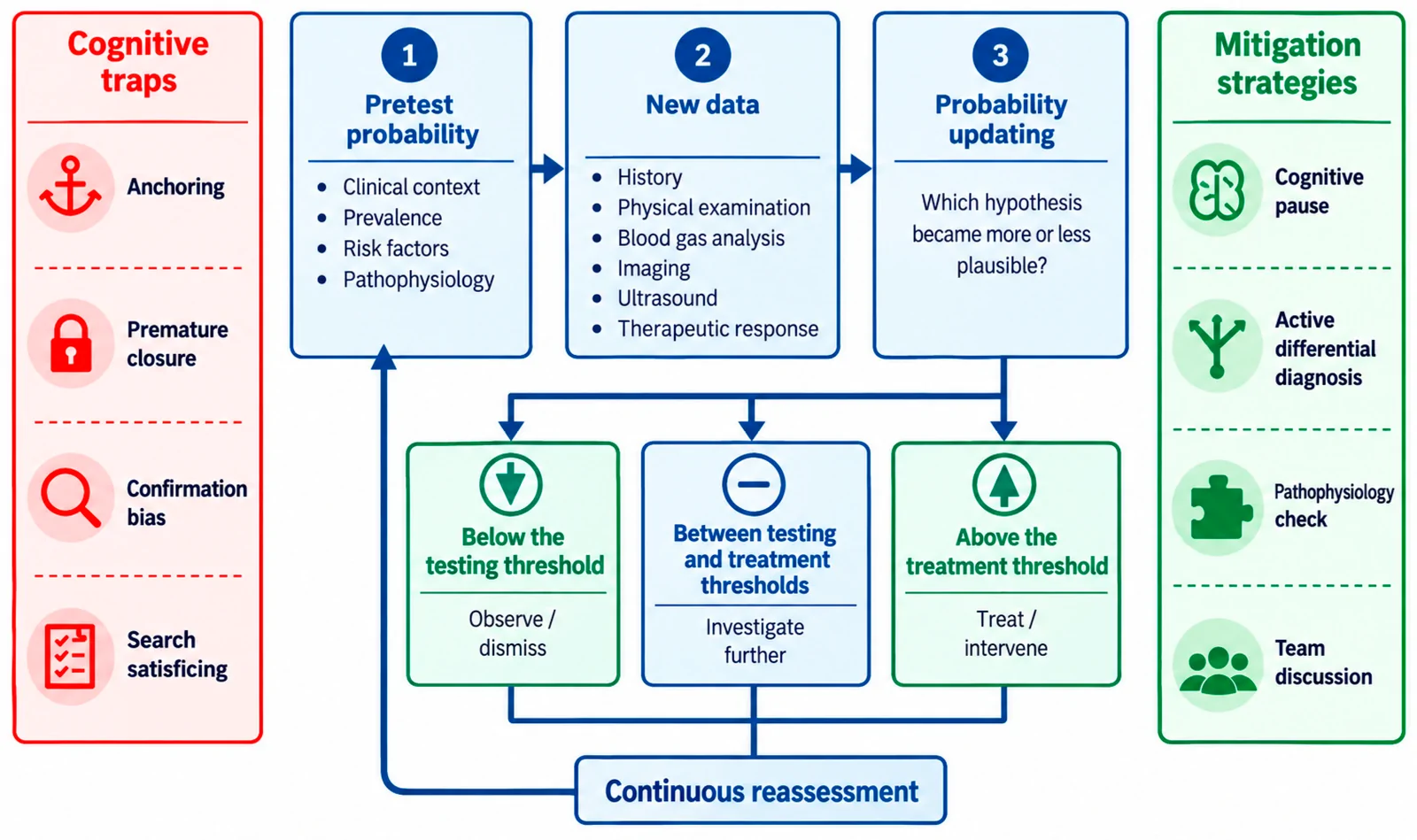
**A conceptual framework for Bayesian reasoning in the intensive care unit.** This figure illustrates a proposed framework for probabilistic clinical reasoning in the ICU. The process begins with an estimate of pre-test probability, based on clinical context, disease prevalence, risk factors, and pathophysiologic understanding. New information, including history, physical examination, blood gas analysis, imaging, ultrasound, and therapeutic response is then incorporated to update the probability of competing diagnostic hypotheses. Depending on the revised probability, the clinician may choose to observe or dismiss the hypothesis (below the testing threshold), pursue further evaluation (between the testing and treatment thresholds), or initiate treatment/intervention (above the treatment threshold). The figure also highlights common cognitive pitfalls (anchoring, premature closure, confirmation bias, and search satisficing) along with the corresponding mitigation strategies (cognitive pause, active differential diagnosis, pathophysiologic cross-checking, and team discussion). Continuous reassessment is essential throughout the process. Because the framework is conceptual, its capacity to reduce diagnostic error has not yet been demonstrated and requires prospective evaluation. Abbreviations: ICU, intensive care unit. Note: This figure was created with the assistance of ChatGPT (OpenAI), a generative artificial intelligence tool, based on conceptual prompts designed by the authors.

Bayesian reasoning makes explicit what good clinicians do intuitively: interpreting each datum considering a prior probability and updating the hypothesis probability in response to new evidence. The same troponin value carries different implications in a young person with atypical chest pain, an older adult with septic shock, and a patient with typical pain, instability, and electrocardiographic changes. Similarly, D-dimer may be uninformative in systemic inflammation in a critically ill patient, but discriminative in low or intermediate pretest probability. The same lactate value may represent hypoperfusion, increased breathing work, a beta-adrenergic effect, hepatic dysfunction, seizure, sepsis, mesenteric ischemia, or mitochondrial dysfunction. Data do not speak for themselves; they modify a situated hypothesis. In the ICU, almost all variables are continuous, unstable, and context-dependent. The probability of pulmonary embolism, myocardial infarction, sepsis, hypovolemia, tamponade, ARDS, ventilator-associated pneumonia, or ventricular dysfunction should be treated as a continuously revisable estimate, not as a fixed category [37]. This approach transcends the excessively dichotomous reading of evidence. Clinical trials and meta-analyses are often dichotomized as “positive” or “negative” based on p-values, yet clinical decision-making requires estimating graded probabilities of benefit, harm, or futility. A study lacking statistical significance may still shift the probability of benefit in a clinically meaningful way; a statistically significant result may be only weakly applicable if the absolute effect is small, the risk of harm is high, or the individual patient differs from the study population. Recent reviews written for clinicians emphasize that Bayesian analysis expresses results as posterior distributions, credible intervals, and probabilities of clinically meaningful effects, thereby favoring an interpretation closer to real-world decisions [38]. In critical care practice, this connects directly to personalized medicine: for this patient, with these characteristics and this initial response, what is the probability of net benefit? Finally, Bayesian thinking ignites clinical practice, adaptive research, and AI. Adaptive platform trials exemplify the capacity to update treatment probabilities as data accrue, allocate patients dynamically to more promising strategies, and stop interventions for superiority, inferiority, or futility. This architecture is compatible with the ICU, where heterogeneity, urgency, and continuous learning make universal conclusions difficult [39]. In decision-support systems, AI would better serve clinical practice if it transcended binary alerts and supported explicit probability updates for the risk of deterioration, the probability of bacterial infection, the likelihood of fluid responsiveness, the risk of extubation failure, and the probability of benefit from a ventilatory strategy. The objective is not to replace judgment, but to make judgment more transparent, calibrated, and revisable (Figure 2). Consider a 74-year-old immunosuppressed woman on day 10 of mechanical ventilation for SARS-CoV-2 pneumonia, who develops refractory hypoxemia. The intensivist’s pre-test estimates include ventilator-associated pneumonia, pulmonary embolism, and fluid overload, each with a distinct mechanism and treatment. An elevated D-dimer and new right ventricular dilation on bedside echocardiography update the probability of pulmonary embolism above the testing threshold, while progressively rising compliance argues against worsening ARDS. When the team consults an AI tool as a cognitive adversary, its differential hypotheses include less common mechanisms such as air embolism and pulmonary tumor embolism. The team did not accept these suggestions automatically. Air embolism was considered unlikely on the grounds that there had been no recent central venous catheter manipulation or removal, no circuit disruption, and no thoracic procedure immediately preceding the deterioration. Pulmonary tumor embolism was deemed improbable given the absence of known malignancy and the absence of radiological findings suggestive of tumor emboli. These alternative mechanisms therefore prompted a targeted search for discordant data rather than further invasive investigation. After this review, and in the absence of contraindications (e.g., recent bleeding or severe thrombocytopenia), the team retained the original plan to anticoagulate for suspected pulmonary embolism, explicitly documenting that the clinician–LLM concordance did not constitute independent confirmation, since both reasoned from the same clinical information and assumptions. The final decision was made by the clinicians and recorded in the medical record. This example illustrates how the Bayesian architecture of Figure 2, pathophysiologic coherence, and governed AI use operate as one integrated cycle.

## 5. Artificial Intelligence and Medical Decision-Making: Impact, Risks, and Efficient Use

AI has entered medicine through multiple domains: image analysis, prognostic prediction, triage, natural language processing, monitoring, clinical documentation, decision support, drug discovery, and medical education. Topol described the convergence of human and AI as a central transformation in high-performance medicine, emphasizing that its value depends on improving human care, not merely automating tasks [40]. In critical care, the appeal is evident. The ICU environment produces continuous and multidimensional data: vital signs, ventilator waveforms, serial laboratory tests, fluid balance, doses of vasoactive drugs, sedation, microbiology, imaging, clinical notes, and adverse events. Systematic reviews of AI in critical care document applications in prediction, monitoring, risk stratification, and decision support, yet highlight problems related to external validation, workflow integration, and actual impact on clinical outcomes [15]. Clinical usefulness depends not only on benchmark accuracy but also on external validity, calibration, explainability, integration into care workflows, safety, equity, governance, and appropriate trust.

A tool with good average performance may still be dangerous in subgroups, in patients outside the training distribution, in contexts with incomplete data, or when its alerts induce excessive intervention. AI in critical care has been described as a relevant opportunity surrounded by obstacles related to data quality, biases, interpretability, implementation, clinical acceptance, and accountability [16]. In acute care medicine, AI systems for complex decision-making face the challenge of operating in environments characterized by high uncertainty, high consequences, and multiple simultaneous objectives [41]. Large language models (LLMs) have transformed the debate because they do not merely classify images or estimate risks; they generate clinical language. They can generate differential diagnoses, explain management decisions, summarize cases, formulate plans, and answer questions.

The AI applications relevant to clinical reasoning can be grouped into three broad approaches, each posing distinct risks. Rule-based clinical decision support systems encode explicit knowledge and are limited by rigidity and maintenance burden. Discriminative machine learning models (e.g., logistic regression, gradient boosting, deep neural networks) estimate risks or classifications from data and excel at prediction, but do not generate clinical language or explanations. Generative large language models produce free-text hypotheses, summaries, and plans, which makes them uniquely interactive but also uniquely susceptible to hallucination, that is, fluent and confident outputs that are factually or clinically incorrect, a serious safety concern in high-stakes settings.

The evidence on LLMs should be interpreted according to the type of study from which it derives. In a randomized clinical trial, access to a large language model did not significantly improve physicians’ diagnostic reasoning compared with conventional resources, and the observed effects depended on how the model was used and integrated with physician assessment [8]. By contrast, another randomized trial found that GPT-4 assistance improved physicians’ performance on patient care tasks [9], and a study in critical illness suggested diagnostic gains when the model was used as an adjunctive tool [10]. Notably, however, these trials involved case- and simulation-based tasks rather than real patients, and none directly measured long-term clinical outcomes.

Evidence from benchmarks and vignettes is more numerous but equally limited: the LLM achieved high performance on expert-level critical care questions in some domains, although with considerable inconsistency and variation across areas [11].

Medical AI should be viewed as cognitive support, not as the final epistemic authority. Large models may lack adequate metacognition; that is, they may not sufficiently recognize the limits of their own knowledge and may continue to provide confident answers when uncertainty should be explicit [12]. Recent evaluations in medical reasoning tasks reinforce that model performance is improving rapidly, but that performance on cases or benchmarks does not automatically translate into real-world clinical safety [13]. This aligns with sociotechnical frameworks proposing that AI should genuinely support the physician’s cognitive and epistemic activities rather than merely optimize performance on decontextualized tests [42]. A practical way to use AI in clinical reasoning is to treat it as a hypothesis generator and cognitive adversary. Instead of asking “what is the diagnosis?”, it is safer to ask: what serious diagnoses could explain these findings? Which data contradict my leading hypothesis? Which pathophysiologic mechanisms could produce refractory hypoxemia in this context? Which adverse effects of current interventions may worsen the patient’s condition? What questions should I ask before accepting this diagnosis? Another efficient approach is to request structure, not a final answer: organize problems by system, list mechanistic hypotheses, identify supporting and opposing data, highlight time-sensitive decisions, and make uncertainties explicit. In this way, AI becomes a tool for problem representation, not an oracle.

In this role, the LLM is best regarded as a generator of additional hypotheses and questions that challenge the ICU team’s reasoning, rather than as an independent second opinion. Because the clinician and the model typically reason from the same clinical dataset, the same incomplete information, and the same underlying assumptions, agreement between them does not constitute independent confirmation; concordance should increase confidence only insofar as the conclusion survived independent pathophysiologic and probabilistic scrutiny. Whether this approach reduces diagnostic errors in the ICU has not been demonstrated and requires prospective evaluation. AI may also help counter biases by recalling rare diagnoses, suggesting unconsidered alternatives, identifying temporal inconsistencies, pointing out that a hypothesis does not explain all the data, and reducing the burden of retrieving dispersed information from the medical record. However, it may also induce new biases: automation bias, algorithmic anchoring, and cognitive complacency. Automation bias occurs when the user accepts a recommendation because it comes from a machine perceived as objective; algorithmic anchoring occurs when the first output organizes all subsequent reasoning; complacency occurs when the tool reduces the effort to think independently. Human-AI interaction studies show that cognitive forcing functions can reduce overreliance. This reinforces the importance of systems that encourage critical thinking rather than automatic acceptance [14]. In critical care, explainability has special importance. Recommendations to increase PEEP, administer fluids, initiate vasopressors, reduce sedation, or anticoagulate cannot be accepted simply because a model suggested them. They must be contestable at the bedside. The intensivist should ask: what mechanism is being assumed? Which data support that mechanism? Which risks were considered? How would the recommendation change if compliance, echocardiography, venous pressure, lactate, or urine output were different? The debate about explainability is particularly relevant in the ICU, where decisions are rapid and potentially irreversible [43]. Guidelines for evaluating explainable AI in medicine reinforce that interpretability is a component of trust, auditability, and clinical applicability [44].

Operational use of AI at the bedside should follow a set of principles, which we present as a proposed framework requiring evaluation rather than a validated protocol. First, the physician should formulate an independent problem representation before consulting AI in high-risk decisions; AI consultation is most likely to help in conditions of diagnostic uncertainty, complexity, or unexpected deterioration, rather than in routine decision-making. Second, the physician should request differential diagnoses rather than a single diagnosis and treat the output as a hypothesis generator, not an oracle. Third, the physician should not treat clinician–LLM agreement as independent confirmation: because the clinician and the model may reason from the same incomplete data and shared assumptions, concordance becomes meaningful only after the conclusion has survived independent pathophysiologic and probabilistic scrutiny. Fourth, the physician should require uncertainty, assumptions, and missing data while recognizing that confidence statements generated by large language models are not reliably calibrated expressions of uncertainty [12]. Fifth, the output should be tested against pathophysiology and rejected when it is mechanistically implausible, ignores a critical missing datum, or conflicts with bedside findings. Sixth, therapeutic recommendations should be cross-checked against reliable sources before implementation. Seventh, clinician–AI disagreement should be resolved by the clinician, documented, and revisited with the team when the stakes are high, with the final decision reflecting the patient’s values, preferences, and goals of care. Eighth, the medical record should make explicit that the final decision was human, contextual, and accountable. Nineth, identifiable data should not be entered into tools that have not been institutionally approved. These bedside principles should be complemented by institutional governance. AI tools should be validated locally and their calibration assessed in the local population before clinical use; models should be clearly versioned, with monitoring for performance drift over time and after model updates; audit trails should record when, how, and on what basis AI outputs informed clinical decisions; and mechanisms for bias assessment, cybersecurity, privacy compliance, and incident reporting should be in place before deployment [14,43,44]. Whether these proposed principles improve safety and outcomes in the ICU requires empirical evaluation. The most useful AI will not be the one that replaces physicians, but the one that improves medical thinking: reducing informational noise, making uncertainty explicit, broadening hypotheses, supporting mechanistic reasoning, and facilitating calibrated decisions (Table 2).

## 6. Training Physicians in Clinical Reasoning with AI and Pathophysiology

Training in clinical reasoning should begin with a simple premise: reasoning well is not an automatic consequence of studying extensively, seeing many patients, or memorizing guidelines. Knowledge is indispensable, but it must be organized, applied, tested, and revised. Traditional training often separates basic science, clinical medicine, epidemiology, and therapeutic decision-making. The result may be learners who know biochemical pathways but do not use them to explain the patient; who know guidelines but do not recognize when they apply poorly; or who use AI to obtain quick answers but do not develop independent judgment. The educational strategies discussed below are supported by variable levels of evidence; where clinical effectiveness has not yet been demonstrated, we explicitly identify them as promising, hypothesis-generating proposals rather than validated approaches. A modern curriculum should integrate biomedical knowledge, illness scripts, problem representation, decision theory, Bayesian thinking, and metacognition. Pathophysiology should be taught as an explanatory tool, not as isolated content. When discussing pneumonia, for example, it is not enough to list etiologic agents and antibiotics. Instead, it is necessary to discuss how alveolar inflammation produces shunts, why this causes hypoxemia, when hypoxemia is disproportionate, how mechanical ventilation alters hemodynamics, when the clinical course suggests a complication, and how to distinguish antibiotic failure from an alternative diagnosis. Empirical evidence indicates that biomedical knowledge contributes to diagnostic reasoning and deep clinical understanding [27,28], and script-based educational methods have shown benefit as formal teaching strategies in a randomized trial [5]. The translation of these findings into a fully integrated curriculum, however, remains an educational design challenge that requires further evaluation. Case-based teaching should be progressive. Typical cases build scripts; comparative cases teach discrimination; complex and ambiguous cases force reassessment, prioritization, and decision-making under uncertainty. The question “what is the diagnosis?” should be replaced by: What is the problem representation? Which hypotheses are likely or dangerous? Which mechanism explains the findings? Which datum contradicts the hypothesis? Which test would change management? Which decision cannot wait? These question frameworks are proposed as instructional scaffolds; their comparative effectiveness has not been formally established. Simulation is a promising strategy for critical care education because it enables training in real-time reasoning without risk to the patient. Scenarios involving refractory shock, sudden hypoxemia, sepsis, tamponade, tension pneumothorax, or ventilator asynchrony should require both action and explanation. Debriefing should examine perceived cues, missed cues, anchoring, premature closure, and pathophysiologic coherence. Although simulation-based education is widely used in critical care training, evidence directly linking simulation to improved clinical reasoning or patient outcomes in the ICU remains limited, and this strategy should be regarded as promising rather than proven.

The use of AI can be incorporated into training as a Socratic tutor, case generator, second-opinion simulator, feedback instrument, and post-case reflection tool. This is a conceptual proposal that has not yet been empirically validated in medical education. Nevertheless, systematic reviews of overreliance on AI dialogue systems warn that substituting AI for active thinking can harm cognitive skill development [17], which argues for cautious introduction. A proposed pedagogical safeguard is to think first, consult afterward, and always criticize. Before using AI, the learner should write the leading hypothesis, alternatives, pathophysiologic mechanism, and initial plan; then consult the AI; finally, critically compare the responses and justify what would change. This rule is hypothesis-generating and requires empirical testing. Training should also include AI literacy, a curriculum component that we propose in light of the tools’ current limitations. Physicians need to understand basic concepts such as training data, external validation, overfitting, algorithmic bias, hallucination, calibration, explainability, privacy, and governance. They do not need to be computer scientists, but they need to know enough to use tools safely. They should understand that a model may be right for the wrong reason, wrong with great confidence, or correct on average yet dangerous in an individual case. Contemporary clinical competence therefore encompasses judgment about intelligent systems. The development of adaptive expertise is especially important. Routine expertise allows familiar problems to be solved well; adaptive expertise allows physicians to deal with new, ambiguous, or unstable problems by reformulating hypotheses and transferring knowledge to unforeseen situations [18,19]. The ICU requires both. Intensivists must rapidly recognize typical situations, but they must also abandon the first hypothesis when the patient does not respond as expected. Pathophysiology is central to this transition because it provides a causal structure for generating new explanations when the script fails. Current assessment methods inadequately capture the dynamics of clinical reasoning and warrant reconsideration. Multiple-choice examinations measure knowledge, but they poorly capture the dynamics of reasoning processes. Proposed alternatives include progressive cases, script concordance tests, written justifications, OSCEs with explicit reasoning, medical record review, error discussion, mechanistic maps, and longitudinal workplace-based observation; some of these tools (e.g., script concordance tests and OSCEs) have established literature [6,7], whereas others require further validation. In residency programs, competence should be observed during rounds, shifts, admissions, handoffs, and critical decisions. Morbidity and mortality conferences should also examine reasoning: What was the initial hypothesis? How was it formed? Which data was ignored? When should it be revised? Was any warning disregarded? If AI was used, did it help or hinder? In the ICU, the goal is to develop situated pathophysiologic reasoning: thinking from the real patient, in real time, under real constraints. The resident should connect mechanisms to decisions: If I increase PEEP, what do I expect to happen to oxygenation, compliance, and arterial pressure? If I administer fluids, which variable should improve? If I start dobutamine, which sign will confirm the benefit? If I reduce sedation, which neurologic or ventilatory risk am I accepting? If AI suggests a hypothesis, which bedside findings make it plausible? The final goal is not to train physicians who reject AI, nor to physicians who depend on it. It is to train physicians capable of thinking with AI, using it to broaden hypotheses, reduce cognitive load, organize data, and stimulate reflection while maintaining clinical responsibility, pathophysiologic judgment, and commitment to the patient. Whether these training approaches achieve these goals is an empirical question that should be addressed in prospective educational research.

## 7. Discussion

This narrative review proposes that improving critical care medicine requires integrating three complementary dimensions: clinical reasoning, which provides the cognitive architecture of decision-making; pathophysiology, which supplies the causal structure needed to interpret biological phenomena; and artificial intelligence, which can organize data, generate hypotheses, expose incongruities, and reduce cognitive burden when thoughtfully designed. We argue that this integration of these three dimensions is not merely additive but synergistic: each dimension compensates for the limitations of the others. Clinical reasoning without pathophysiologic grounding risks superficial pattern matching; pathophysiology without probabilistic reasoning risks overconfidence in mechanistic explanations; and AI without critical clinical supervision risks automation bias and epistemic complacency. Generative models, in turn, may produce fluent but factually incorrect outputs (hallucinations) that are often difficult for clinicians to detect and, if unrecognized, can undermine therapeutic decisions [45]. The relationships among these dimensions deserve explicit attention. Clinical reasoning supplies the overall architecture within which hypotheses are generated, tested, and revised. Pathophysiology provides the causal grammar that determines which hypotheses are biologically coherent and which interventions are mechanistically aligned. Bayesian reasoning provides the probabilistic grammar that determines how new data should update the probability of each hypothesis and where testing and treatment thresholds lie. AI, in turn, can feed this cycle by organizing data, suggesting hypotheses, and making uncertainty explicit: provided its outputs are subjected to the same pathophysiologic and probabilistic scrutiny applied to any other source of information. Consistently, when the clinician and the LLM reach the same conclusion, this agreement should not be interpreted as independent confirmation, because both may reason from the same incomplete data and shared assumptions; concordance becomes meaningful only after the conclusion has survived independent pathophysiologic and probabilistic scrutiny. Human factors and patient-centered values are integral to this cycle: workload, fatigue, interruptions, and team communication shape the conditions under which reasoning occurs, and the patient’s values, preferences, and goals of care ultimately define which decisions are appropriate.

This framework also addresses a gap in the existing literature, which has largely treated clinical reasoning, pathophysiology, and artificial intelligence as separate fields. Prior reviews have examined AI applications in the ICU, the cognitive science of diagnostic error, and the role of biomedical knowledge in reasoning, but few have proposed an operational integration linking a Bayesian decision architecture, mechanistic coherence, and governance of AI at the bedside [15,16,41]. Consistent with prior work, we emphasize that diagnostic errors rarely result from a single cognitive failure but emerge from the interaction of cognitive biases, system factors, and case characteristics. Our contribution is therefore a conceptual synthesis that can serve as a testable foundation for future research and curriculum design, rather than a claim of demonstrated clinical benefit.

### Limitations

This review has inherent limitations. As a narrative review, the selection of studies relied on the authors’ judgment of relevance, which may introduce selection bias, and no formal risk-of-bias assessment or quantitative synthesis was performed. The integrative framework proposed is conceptual and has not yet been empirically validated in clinical practice. Moreover, given the rapidly evolving nature of AI in medicine, some evidence reviewed may become outdated quickly. Finally, although focused on critical care, much of the underlying literature derives from broader medical settings, and its direct applicability to the ICU should be interpreted with caution.

## 8. Conclusions

Given the narrative and conceptual nature of this review, the following conclusions should be interpreted as proposals that warrant empirical evaluation rather than definitive statements. Clinical reasoning remains at the center of medicine, since medical practice continues to involve interpretation and decision-making under uncertainty. In fact, its evolution reflects a transition from anatomoclinical recognition to hypothetico-deductive, probabilistic, cognitive, educational, and sociotechnical models. Moreover, each stage added something essential: observation, hypothesis, probability, decision, metacognition, teamwork, and technology. We argue that medical errors resulting from poor reasoning occur when hypotheses are accepted too early, discordant data are ignored, mechanisms are not tested, tests are interpreted without pretest probability, systems fragment information, and technologies are used without critical supervision. Therefore, preventing such errors is likely to require knowledge, structure, culture, feedback, and epistemic humility. We also propose that pathophysiology should be regarded as a core component of individualization, since it can help clinicians to move beyond the diagnostic label and explain why the patient is unstable, why an intervention worked or failed, why a clinical course is unexpected, and why a recommendation may be inappropriate despite appearing statistically plausible. In critical care, where mechanisms overlap and decisions are time-sensitive, mechanistic reasoning appears particularly valuable; however, mechanistic plausibility alone does not confirm diagnostic or therapeutic correctness. Similarly, we suggest that Bayesian thinking makes explicit the probabilistic nature of medical decision-making. Clinical decisions are not isolated acts of classification, but continuous processes of hypothesis updating. This logic may be especially powerful in the ICU, where probabilities change rapidly, therapeutic responses have diagnostic value, and average effects may not represent the individual patient. Finally, we propose that AI inaugurates a new chapter in critical care medicine. On the one hand, it may help organize data, generate hypotheses, provide a second opinion, reduce cognitive load, and support decisions; on the other hand, it may also amplify errors through hallucination [45], opacity, bias, automation, and false confidence. We therefore argue that its value will depend less on replacing physicians and more on improving physicians’ reasoning. In sum, we propose that the best practice may emerge from a disciplined combination of human intelligence, deep pathophysiologic understanding, probabilistic reasoning, and critically governed artificial tools.

## 9. Future Directions

The conceptual integration of clinical reasoning, pathophysiology, and AI proposed in this review establishes a foundation for several key research priorities:

**Empirical validation of the integrative framework.** Prospective studies are needed to evaluate whether implementing this tripartite decision architecture in the ICU improves measurable clinical metrics, such as diagnostic accuracy, time to appropriate targeted therapy, and reduction in preventable diagnostic errors.

**Design and evaluation of reasoning-oriented AI tools.** Rather than relying on predictive models that function as opaque diagnostic “oracles,” future software design should prioritize tools that structure problem representation, expose data incongruities, and explicitly communicate uncalibrated uncertainty to the clinician.

**Quantifying AI-induced cognitive biases in practice.** Automation bias, algorithmic anchoring, and cognitive complacency are recognized risks of human-AI interaction, yet they remain poorly characterized in the high-stakes, time-pressured ICU environment.

**Long-term evaluation of AI-integrated training.** Curriculum studies should assess whether Socratic AI-assisted training enhances adaptive expertise and mechanistic reasoning without causing skill decay or overreliance among trainees.

**Implementation of governance and safety standards.** Translating theoretical principles into practice requires establishing concrete institutional protocols for local model validation, performance drift monitoring, audit trails, and privacy compliance.

**Integration with Learning Health Systems.** Linking Bayesian clinical reasoning with adaptive trial designs and continuous real-world data collection will allow bedside decision-making to dynamically inform and refine critical care evidence bases.

## Figures and Tables

**Table 1:** Summary of principal studies informing the review.

Study (Ref.)	Design	Population/ Setting	Main Outcome	Limitation
**Clinical reasoning & education**				
Moghadami et al. [5], 2021	Randomized controlled trial	Undergraduate medical students	Illness-script method improved clinical reasoning scores vs. conventional teaching	Single-center; short-term assessment
Norman [6], 2005	Narrative review	Clinical reasoning research	Expert reasoning relies on organized knowledge networks, not linear rules	Conceptual synthesis
Eva [7], 2005	Narrative review	Medical education	Describes what teachers need to know about clinical reasoning and its assessment	Conceptual synthesis
**Artificial intelligence**				
Goh et al. [8], 2024	Randomized clinical trial	Physicians, case-based tasks	LLM assistance did not significantly improve diagnostic reasoning vs. conventional resources; effects depended on integration	Simulation-based, not real patients
Goh et al. [9], 2025	Randomized controlled trial	Physicians, patient-care tasks	GPT-4 assistance improved performance on patient-care tasks	Simulation-based
Wu et al. [10], 2025	Comparative study	Complex critical-illness cases	LLM as adjunct improved clinicians’ diagnostic performance	Case-based; single model
Workum et al. [11], 2025	Benchmark study	Expert-level critical care questions	High performance in some domains, with inconsistency across areas	Benchmark, not clinical outcomes
Griot et al. [12], 2025	Evaluation study	Medical reasoning tasks	LLMs lack essential metacognition for reliable medical reasoning	Benchmark-based
Brodeur et al. [13], 2026	Evaluation study	Physician-level reasoning tasks	Rapidly improving performance; not yet shown to translate to clinical safety	Benchmark, not real-world
Buçinca et al. [14], 2021	Experimental study	Human–AI decision tasks	Cognitive forcing functions reduce overreliance on AI	Laboratory setting
**Systematic reviews/critical care**				
van de Sande et al. [15], 2021	Systematic review	AI in the ICU	Applications in prediction/monitoring/decision support; gaps in external validation and clinical impact	Heterogeneous studies
Pinsky et al. [16], 2024	Expert review	AI in critical care	Opportunity surrounded by obstacles (data quality, bias, interpretability, accountability)	Expert opinion
Zhai et al. [17], 2024	Systematic review	Students using AI dialogue systems	Overreliance may harm cognitive skill development	Educational, not clinical
**Adaptive expertise**				
Ng et al. [18], 2022	Conceptual review	Health professions education	Combines adaptive expertise and critically reflective practice	Conceptual
Pelgrim et al. [19], 2022	Overview of reviews	Educational/workplace settings	Adaptive expertise and adaptive performance framework	Overview, not primary data

**Table 2:** Operational principles and governance safeguards for the safe use of AI at the bedside.

**Principle**	**Description**
**1.** **Independent problem representation first**	Formulate the clinical picture before consulting AI in high-risk decisions. Reserve AI consultation for situations of diagnostic uncertainty, complexity, or unexpected deterioration.
**2.** **Request differential diagnoses, not a single answer**	Ask for a hierarchy of plausible hypotheses and treat the output as a hypothesis generator, not an oracle.
**3.** **Do not treat clinician–LLM agreement as** **independent confirmation**	The clinician and the model may reason from the same incomplete data and shared assumptions; concordance becomes meaningful only after the conclusion has survived independent pathophysiologic and probabilistic scrutiny.
**4.** **Require explicit uncertainty**	Demand that assumptions, missing data, and confidence limits be stated, while recognizing that confidence statements generated by large language models are not reliably calibrated expressions of uncertainty.
**5.** **Test the response against pathophysiology**	Verify mechanistic plausibility before accepting a recommendation; reject outputs that are mechanistically implausible, ignore a critical missing datum, or conflict with bedside findings.
**6.** **Verify high-risk therapeutic recommendations**	Cross-check significant-risk suggestions against reliable sources before implementation.
**7.** **Resolve disagreement transparently**	Clinician–AI disagreement should be resolved by the clinician, documented, and revisited with the team when the stakes are high. The final decision should reflect the patient’s values, preferences, and goals of care.
**8.** **Keep the decision human and accountable**	Document that the final decision was human, contextual, and accountable.
**9.** **Protect identifiable data**	Do not enter patient-identifiable data into tools that have not been institutionally approved.
**Governance Safeguards**	**Description**
**Validate locally and monitor over time**	Validate AI tools in the local population and assess calibration before clinical use; version models clearly and monitor for performance drift after updates.
**Keep audit trails and ensure safety mechanisms**	Record when, how, and on what basis AI outputs informed clinical decisions; implement bias assessment, cybersecurity, privacy compliance, and incident reporting before deployment.

Footnote: These principles synthesize recommendations from the literature on cognitive biases in human–AI interaction [14], explainable AI in medicine [43,44], overreliance on AI dialogue systems [17], and the limits of LLM metacognition and confidence calibration [12]. They are proposed as a framework for evaluation; their effectiveness has not yet been demonstrated in controlled clinical studies, and they should be interpreted in light of local institutional policies and the specific clinical context.

## Data Availability

No new data were created or analyzed in this study. Data sharing is not applicable to this article.

## Abbreviations

AI: artificial intelligence; ARDS: acute respiratory distress syndrome; EBM: evidence-based medicine; ICU: intensive care unit; LLM: large language model; PEEP: positive end-expiratory pressure.
